# Network-Wide GIS Mapping of Cycling Vibration Comfort: From Methodology to Real-World Implementation

**DOI:** 10.3390/s25196185

**Published:** 2025-10-06

**Authors:** Jie Gao, Xixian Wu, Zijie Xie, Liang Song, Shandong Fang

**Affiliations:** 1 School of Civil Engineering and Architecture, East China Jiaotong University, Nanchang 330013, China; 2 Jiangxi Provincial Key Laboratory of Traffic Infrastructure Safety, East China Jiaotong University, Nanchang 330013, China; 3 College of Civil Engineering and Architecture, Xinjiang University, Urumqi 830017, China; 4 School of Ecology and Environment, Xinjiang University, Urumqi 830017, China; 5 School of Traffic and Transportation Engineering, Xinjiang University, Urumqi 830017, China; 6 Highway Development Center of Xinjiang Uygur Autonomous Region, Urumqi 830000, China

**Keywords:** cycling-induced vibration, vibration measurement, comfort evaluation, cycling comfort map

## Abstract

Cycling-induced vibration significantly affects riding comfort, with road surface conditions and vehicle type identified as primary contributing factors. This study developed a vibration measurement system based on ISO 2631-1, and proposed a method for generating cycling comfort maps grounded in vibration severity levels. Field measurements on 30 campus roads in Nanchang, China, used a Mountain Bike, Shared E-bike, and Shared Bicycle. Triaxial acceleration data were collected to evaluate vibration exposure, and comfort levels were classified to produce spatially resolved maps. Results show the proposed system has strong stability and adaptability across urban environments. The maps effectively captured vibration intensity variations along road segments. Among the three vehicle types, Mountain Bikes showed the lowest vibration exposure, with approximately 90% of segments rated as comfortable. Shared E-bike exhibited moderate vibration levels, with 42% of segments deemed uncomfortable, while Shared Bicycles experienced the highest vibration, with 80% of routes potentially inducing discomfort and only 1% meeting comfort standards. This study offers a framework for objective acquisition and visualization of cycling vibration data. The developed system and mapping method provide tools for assessing vehicle vibration, guiding route selection, and offer potential value for road quality monitoring.

## 1. Introduction

The rapid increase in motor vehicle numbers has exacerbated traffic congestion and environmental pollution, posing unprecedented challenges to urban transportation. Although the adoption of new energy technologies has mitigated environmental pollution to some extent [1], traffic congestion remains a severe issue, and the demand for sustainable urban development is becoming increasingly prominent. In this context, cycling has emerged as a low-carbon and efficient alternative to alleviate urban traffic pressure. Research indicates that well-developed road infrastructure significantly enhances the cycling experience [2,3], while complex traffic environments may negatively impact cyclists’ safety and comfort [4]. Additionally, long-term cycling has been shown to improve cardiovascular health and enhance physical fitness [5,6], although excessive vibration can adversely affect cycling comfort and human health [7,8]. Studies have demonstrated that cycling vibration is influenced by multiple factors, including cycling speed, road quality, and vehicle type [9,10,11,12]. Asphalt pavements generally exhibit lower vibration levels, whereas concrete surfaces tend to generate higher vibrations [3]. Therefore, optimizing road conditions and reducing cycling vibrations not only enhances riding comfort but also encourages greater public participation in cycling, thereby fostering sustainable urban mobility [13,14].

In vibration testing, sensor-based data acquisition is a widely adopted approach [9,15,16,17,18]. By mounting accelerometers, GPS modules, and other sensing devices on bicycles, vibration signals during riding can be effectively captured [8,19,20]. Additionally, some studies incorporate camera modules to synchronously record environmental conditions, facilitating data retrospection and vibration analysis. For cycling comfort evaluation, various methodologies have been employed. The Dynamic Comfort Index (DCI), ranging from 0 to 1 and inversely correlated with riding comfort, has been used for quantitative assessments [21]. Another prevalent approach involves computing the root mean square (RMS) value of vertical (*Z*-axis) vibrations to assess vibration intensity during cycling [9,22]. Moreover, the weighted RMS acceleration ($a_{wv}$) has been extensively applied in comfort evaluation and validated across multiple studies [23]. Recent research has also leveraged See. Sense smart bike lights for comfort assessment, conducting comparative analyses with DCI and other metrics. The results indicate the feasibility of this method for real-world applications [24]. Accelerometers and GPS modules have been extensively employed in bicycle vibration testing. Although their effectiveness has been demonstrated in numerous studies, existing methods still exhibit certain limitations and shortcomings [25]. The testing process remains cumbersome, and discrepancies in data timestamps introduce errors, compromising measurement accuracy. Consequently, the development of a high-efficiency, high-precision cycling vibration testing system capable of ensuring accurate data synchronization and systematically evaluating road-induced vibrations has emerged as a critical research focus. Additionally, GIS technology has been widely used in urban planning and transportation sectors for tasks such as traffic flow analysis, infrastructure planning, and optimal route selection [26,27].

This study developed a cycling vibration testing system by integrating vibration accelerometers, GPS modules, and Raspberry Pi. The system achieves high-precision synchronous acquisition of acceleration data and geospatial information. It effectively eliminates data errors caused by inconsistent start times in previous research. This significantly improves measurement accuracy. Using field-collected cycling vibration data and Geographic Information System (GIS) technology, a cycling vibration comfort map was created. The research aims to establish a standardized and quantifiable evaluation framework for cycling vibration comfort. It provides practical route selection guidance for cyclists.

## 2. Experiments

### 2.1. Cycling Vibration Dynamic Testing Hardware System

#### 2.1.1. System Composition

**Figure 1:**
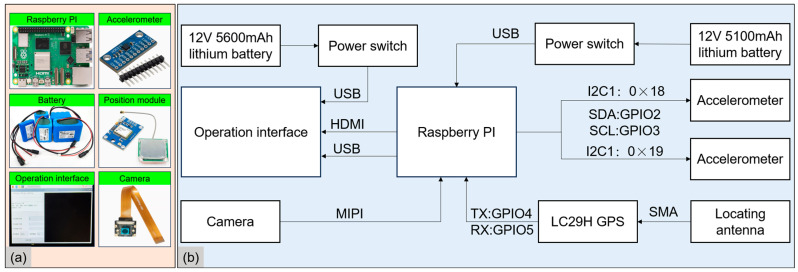
Cycling vibration dynamic testing hardware system. (**a**) Diagram of the module (source: product manual); (**b**) Schematic block diagram.

**Figure 2:**
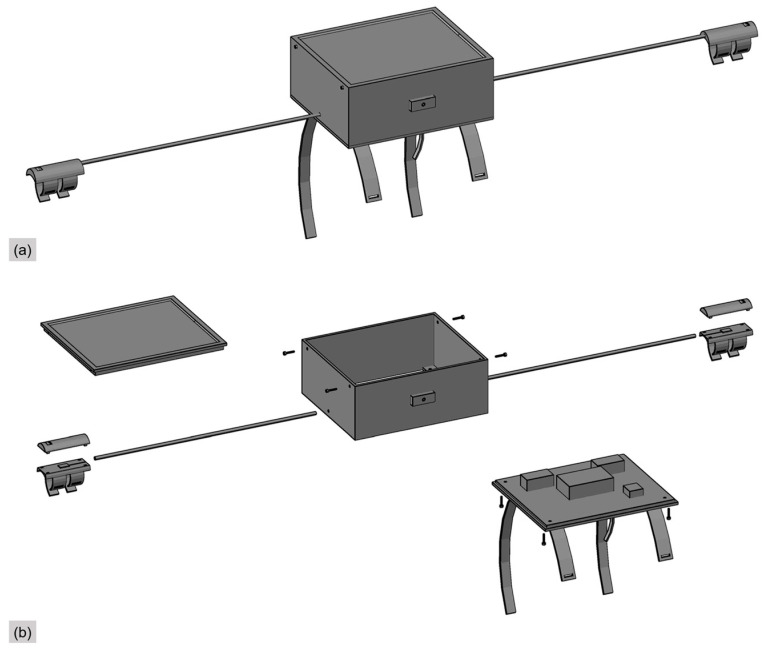
Schematic diagram of instrument integration. (**a**) Overall schematic diagram of the instrument; (**b**) Exploded view diagram.

Currently, there is a lack of dedicated instruments designed exclusively for cycling vibration testing, and many existing studies rely on the integration of multiple devices to capture relevant data [9,15,16]. However, such an approach is not only cumbersome but also prone to errors resulting from timestamp discrepancies between different instruments. To address these challenges, this study developed an integrated dynamic cycling vibration testing system. The proposed hardware system features one-touch start and stop functionality, parameter configuration, and real-time data display, significantly streamlining the testing process while enhancing data acquisition accuracy and consistency. The system is mounted on the bicycle’s handlebars and comprises 6 core modules: a microcontroller, a triaxial vibration accelerometer, a GPS module, a camera module, a touchscreen interface, and a power management unit. The system is illustrated in Figure 1, and the overall integration of the instruments is shown in Figure 2.

#### 2.1.2. Module Parameters

According to the ISO 2631-1 [28], the frequency range of whole-body vibration in humans is typically defined as 0.5 Hz to 80 Hz. To ensure data accuracy and prevent aliasing during vibration testing, the sampling frequency must be at least twice the Nyquist frequency, i.e., no less than 160 Hz. A higher sampling frequency not only enables precise capture of vibration signal details but also ensures reliable data acquisition under complex road conditions. Furthermore, vibration signals often contain transient shock components, such as short-duration high-frequency vibrations induced when traversing potholes, cracks, or speed bumps. If the sampling rate is insufficient, critical information may be lost, compromising the integrity of vibration characteristics. In this study, to achieve high temporal resolution for geolocation data, each second’s variations are precisely captured, thereby enhancing the accuracy of comfort assessments. Accurate recording of the cycling trajectory and maintaining a high degree of synchronization between vibration and location data are essential for precise comfort evaluation. Thus, the positioning module must operate at a minimum sampling frequency of 1 Hz to ensure accurate acquisition of geospatial data per second. A higher update rate in GPS tracking helps mitigate path deviations caused by signal drift, particularly in urban environments where buildings may obstruct satellite signals. Considering these requirements, the accelerometer module’s sampling frequency is set to 400 Hz, while the positioning module is configured at 1 Hz. To ensure the overall stability of the data acquisition system, the technical parameters of each module must be rigorously selected. The seamless integration and coordination of different hardware components directly influence data integrity and accuracy. Therefore, hardware selection must balance real-time performance, precision, and power consumption. The detailed specifications and functionalities of the hardware modules employed in this study are presented in Table 1.

In accordance with ISO 2631-1 standards, band-pass filtering was applied to the whole-body vibration signals to ensure analytical precision. A high-pass filter with a cutoff frequency of 0.5 Hz was employed to eliminate low-frequency interference below this threshold, while a low-pass filter with a cutoff frequency of 80 Hz was utilized to suppress high-frequency noise. This frequency range (0.5–80 Hz) encompasses the spectrum to which the human body is most sensitive, thereby conforming to the recommendations outlined in ISO 2631-1.

### 2.2. Cycling Vibration Test

#### 2.2.1. Study Area

**Figure 3:**
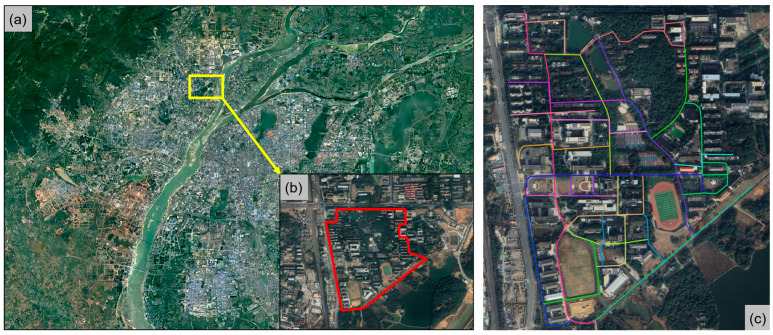
Test area and sampling roads. (**a**) Downtown of Nanchang City; (**b**) Tested position; (**c**) Test road.

This study tests the hardware system on various road surfaces to validate its stability and universality. The tests are conducted under minimal traffic and pedestrian flow to mitigate external interference. Urban road networks, while dense, predominantly feature high-quality asphalt surfaces, presenting a limited variety for testing. Additionally, the dense vehicular and pedestrian traffic can distract cyclists, potentially skewing the test results. Rural roads offer a diverse range of surfaces but are too dispersed to allow for concentrated testing efforts. Consequently, this study opts for the campus roads of East China Jiaotong University as the testing site. Located in the Economic and Technological Development Zone of Nanchang, China (as shown in Figure 3a,b), the campus boasts a dense road network with a mix of asphalt and cement surfaces of varying quality, effectively capturing the vibration characteristics of cycling across different road conditions. Moreover, during specific timeframes, the area experiences relatively low traffic and pedestrian volumes, providing the requisite quiet environment for testing. Given the density and complexity of the road network within the campus, to prevent redundant testing and reduce potential errors therefrom, this study meticulously classified and planned the testing routes in advance, ensuring the independence and accuracy of each test segment, as depicted in Figure 3c.

#### 2.2.2. Test Procedure

**Figure 4:**
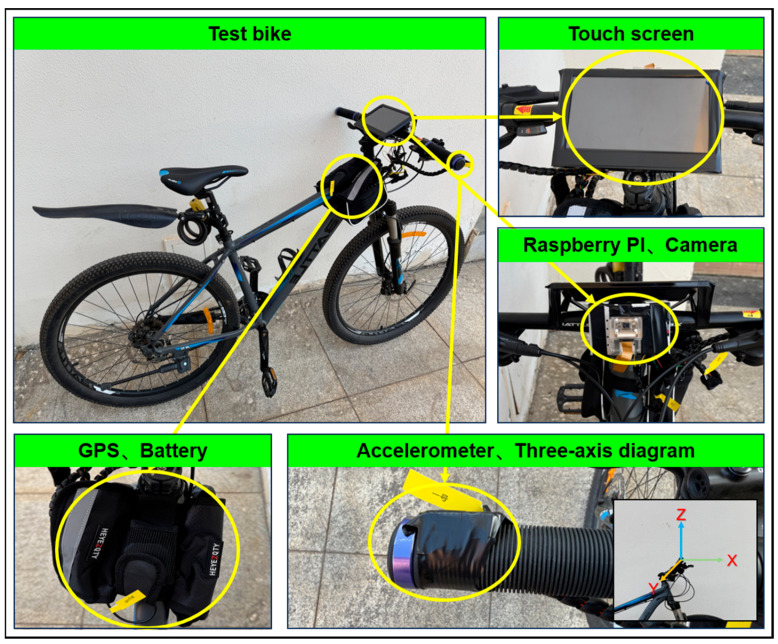
Dynamic cycling vibration testing system.

This study employs an on-site field testing methodology. The vibration data collector was a healthy 25-year-old male, 180 cm tall and weighing 75 kg, who performed the tests in a bent-over posture. He is an avid cycling enthusiast with extensive riding experience, having accumulated over 15,000 km in the past decade. The testing was conducted in three sequential steps. First, the environmental conditions of the testing site are assessed. Cyclists experience heightened stress when navigating through complex traffic environments [4], thus, it is imperative to ensure minimal vehicular and pedestrian traffic to substantially reduce external interference affecting the test results. Second, the installation and calibration of the equipment are carried out. As illustrated in Figure 4, the positioning module, microcontroller, and touch display are securely mounted on the handlebars, with vibration-damping foam placed at contact points to prevent potential damage from excessive vibrations during the test. The accelerometer is installed at the point where the hand contacts the handlebar and is precisely calibrated using a level to ensure its *z*-axis is perpendicular to the ground, *x*-axis aligns with the direction of travel, and *y*-axis points horizontally. Finally, the instruments are activated, and relevant parameters are adjusted as necessary to commence testing. To accurately reflect vibration intensity, the starting and ending points of the cycling test are set beyond the actual test segment to ensure cyclists reach and maintain the predetermined speed (16–20 km/h as specified in this study) upon entering the test segment until exiting the endpoint. Throughout the test, cyclists must adhere to the predetermined route to maintain consistency in the test path, avoiding handlebar sway and abnormal acceleration or deceleration behaviors that could skew the results. Each test exceeds 60 s in duration. Following test completion, the data quality is rigorously assessed. Any irregularities, including signal dropout or atypical measurements, render the dataset invalid and necessitate a retest.

#### 2.2.3. Data Processing

In accordance with the International Organization for Standardization ISO2631-1, the assessment of human vibration exposure is conducted by analyzing vibration signals along the x, y, and z axes, utilizing the Root Mean Square (RMS) acceleration as a quantitative descriptor. The RMS acceleration requires frequency-weighted processing, as recommended by the ISO standard, and is subjected to narrowband filtering to derive the weighted frequency RMS acceleration value, unit: m/s^2^. Vibration signals along the *x*, *y*, and *z* axes, captured via a triaxial accelerometer, are denoted as $a_{wx}$, $a_{wy}$, and${\;a}_{wz}$, respectively, with units of m/s^2^. The calculation formula is provided in Equation (1).

(1) $$a_{wi}={\left[\frac{1}{T}\int_{0}^{T}a_{wi}^{2}(t)dt\right]}^{1/2};i=x,y,z$$

In the equation, $a_{w}\left(t\right)$ represents the time history of the weighted acceleration, with the unit of m/s^2^, and (*T*) denotes the duration of the test, measured in seconds (s).

The total weighted Root Mean Square (RMS) acceleration $a_{wv}$ comprehensively integrates the vibration intensities along the *x*, *y*, and *z* axes, and its calculation is expressed by Equation (2).

(2) $$a_{wv}=\sqrt{a_{wx}^{2}+a_{wy}^{2}+a_{wz}^{2}}$$

The ISO 2631-1 delineates the thresholds for vibration intensity and their potential impact on comfort, as detailed in Table 2. This standard furnishes a scientific basis for the assessment of vibration comfort and is extensively applied in the research and evaluation of various vibration environments.

### 2.3. Comfort Mapping for Cycling

#### 2.3.1. Comfort Level Classification

The vibration values per se cannot directly reflect the comfort level of cycling, and even when combined with latitude and longitude data, it is challenging for cyclists to intuitively identify the specific road conditions of a given section. To address this issue, coupling vibration values with latitude and longitude data and visualizing them on a map is an effective solution. Therefore, this study, in accordance with the ISO 2631-1, employs Geographic Information System (GIS) technology and utilizes QGIS Desktop 3.34.12 to create a cycling vibration comfort map, which comprehensively and meticulously displays the distribution of vibration comfort across a 10.5 km test route. In this manner, cyclists can gain a more intuitive understanding of the comfort levels of different road segments, thereby making more informed cycling decisions.

Furthermore, to achieve a more nuanced representation of cycling vibration comfort, this study further refined the classification of vibration intensity and potential human comfort levels based on the ISO 2631-1. The research employs three colors—green, yellow, and red—to represent different levels of comfort. Specifically, green indicates comfortable cycling sections, yellow denotes sections with poorer but still acceptable comfort, and red marks sections with extreme discomfort, which cyclists should avoid whenever possible. To ensure the precision of the comfort assessment, each major color category is subdivided into ten levels based on vibration intensity, ranging from light to dark. The gradient shades of green, yellow, and red are assigned numerical values from 1 to 10 to avoid scenarios where different vibration values might span multiple comfort levels, thereby enhancing the accuracy of the comfort assessment. The detailed criteria for the comfort level classification are presented in Table 3.

#### 2.3.2. Map Generation

**Figure 5:**
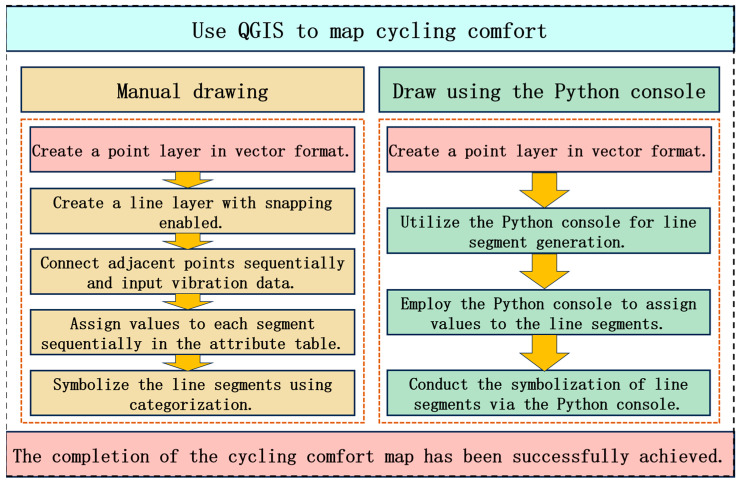
Use QGIS to draw the process of cycling comfort map.

In this study, QGIS Desktop 3.34.12 was employed for the generation of cycling comfort maps. Two primary methods are available for map rendering: manual drawing and automated scripting via the Python 3.13 console. While the manual approach is relatively straightforward, it becomes cumbersome and inefficient when handling large-scale datasets. Moreover, it is susceptible to human-induced errors, which may lead to cumulative inaccuracies. In contrast, Python-based automation enables high-throughput processing, making it well-suited for large datasets by offering superior efficiency and minimized error propagation. Given the substantial data volume involved in this study, Python scripting was selected for map generation, with route segmentation refined at a per-second resolution. This approach aims to demonstrate the variations in vibration rather than to directly evaluate vibration levels. The specific mapping workflow is illustrated in Figure 5. During map rendering, data beyond the designated start and end points of the test segment must be trimmed, ensuring that only the relevant road section is retained for analysis.

According to ISO 2631-1, whole-body vibration assessment must account for both inter-individual variability and temporal fluctuations. This study conducted a preliminary analysis based on data from a single cyclist to validate the feasibility of the vibration test and comfort mapping methodology. To examine the temporal stability of the measurements, vibration data were repeatedly collected from the same cyclist traveling at identical speeds along the same route on different dates. Results indicated variability in vibration magnitudes across trials, whereas comfort evaluations remained consistent, likely reflecting influences from changing road conditions and environmental factors such as temperature, humidity, and wind speed. Although physiological differences and cycling styles among individuals are known to affect vibration responses, constraints on time and resources precluded further investigation in this study. Future research will expand the sample size to include participants of diverse ages, body types, and riding behaviors, thereby enhancing the representativeness and generalizability of the findings.

## 3. Results and Discussion

### 3.1. Reliability Verification

#### 3.1.1. Cycling Trajectory and Speed Validation

**Figure 6:**
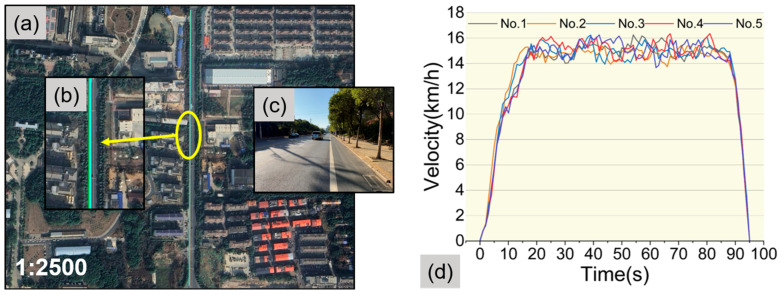
Cycling trajectories and speeds from five rides. (**a**) Test location; (**b**) Cycling trajectories from five rides; (**c**) Reference white line on the test route; (**d**) Cycling speed recorded by the hardware system.

To validate the performance of the hardware system and the reliability of the rider’s cycling activity, this study conducted verification tests on cycling trajectory, cycling speed, and vibration stability. For the trajectory and speed verification tests, the cyclist performed five consecutive rides on the same test route (as shown in Figure 6a). During each ride, the cyclist used the white line on the road as a reference marker (as illustrated in Figure 6c) and followed the line to maintain consistency. By analyzing the trajectory and speed data from the five rides, the performance of the hardware system and the reliability of the rider’s cycling activity were indirectly assessed. The results presented in Figure 6b demonstrate that the five cycling trajectories exhibit a high degree of overlap, indicating that the hardware system achieves high precision in recording cycling trajectories. Furthermore, the results in Figure 6d reveal that the speed data from the five rides are remarkably consistent, suggesting that the rider was able to skillfully operate the bicycle and effectively maintain the speed within the predetermined range. These findings robustly demonstrate that the hardware system is capable of accurately and promptly recording cycling trajectory and speed data, meeting the requirements of the test. This further validates the reliability of both the hardware system and the rider in practical testing scenarios.

#### 3.1.2. Vibration Stability Verification

**Figure 7:**
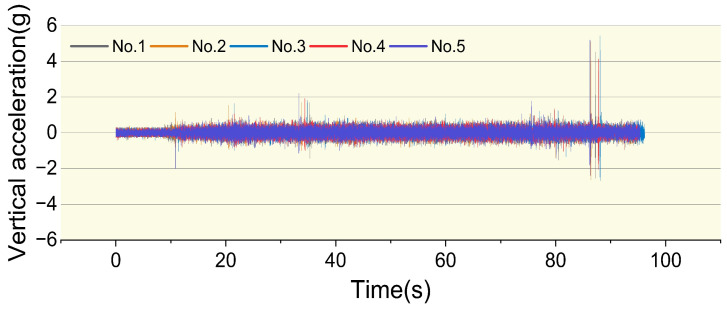
Raw vibration signals.

To assess the reliability of the vibration data, while testing cycling speed and trajectory, the accelerometer simultaneously recorded vibration data at a sampling frequency of 400 Hz during the five rides. The vibration data collected by the hardware system must exhibit sufficient stability to ensure its reliability in practical applications. Figure 7 presents the unprocessed raw vibration signals in the *z*-axis obtained by the cycling vibration dynamic testing hardware system during the five rides.

**Figure 8:**
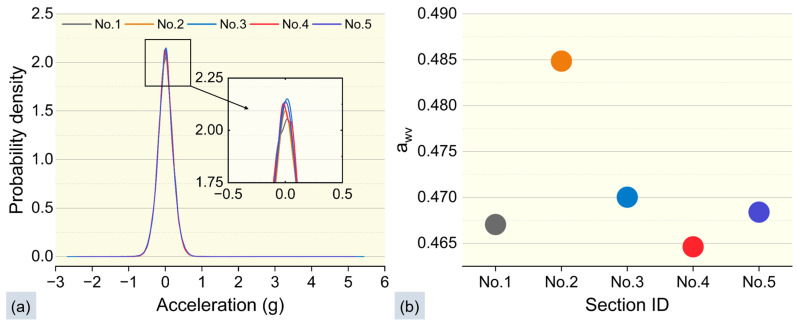
Vibration Stability Across Five Tests. (**a**) Vibration probability density distribution curves using the Kolmogorov–Smirnov test; (**b**) Vibration intensity $a_{wv}$.

To analyze the collected vibration data, the Kolmogorov–Smirnov test was employed to obtain the probability density distribution curves of the vibrations. The results presented in Figure 8a demonstrate that the vibration acceleration values from the five rides follow a normal distribution, and the respective normal distribution curves exhibit a high degree of overlap, further validating the reliability of the hardware system in capturing vibration acceleration values. Additionally, Figure 8b presents the results of the vibration intensity values calculated for the five rides (the calculation method is detailed in Section 2.2.3), revealing that the errors in all five tests are below 0.1 m/s^2^.

### 3.2. Field Testing

#### 3.2.1. Road Conditions and Environment

**Figure 9:**
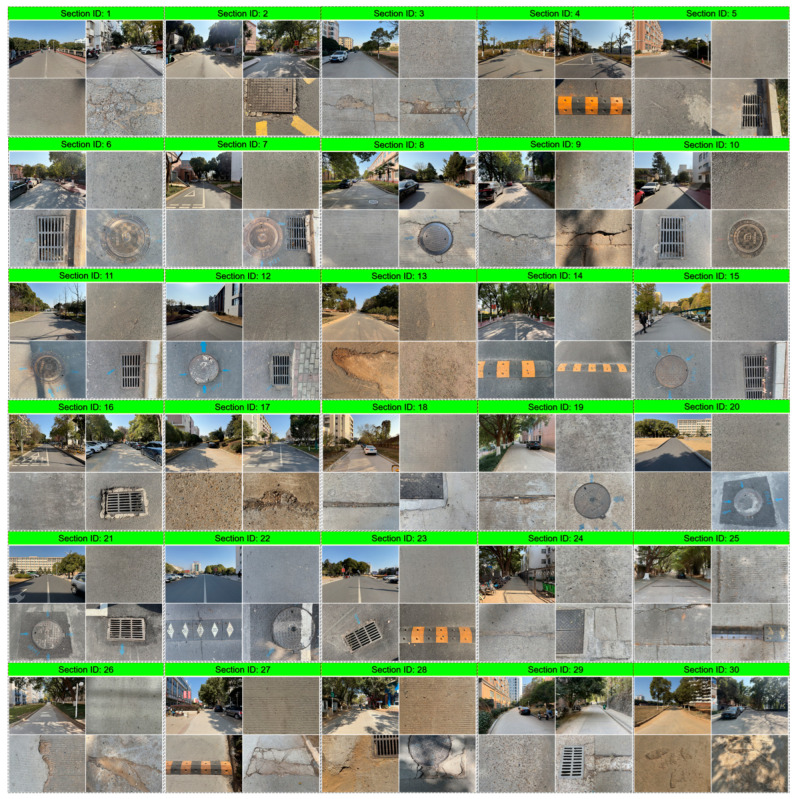
Surrounding environment and road surface conditions of the tested segments.

In this study, a total of 30 road segments were tested, covering a combined length of approximately 10.5 km, as illustrated in Figure 9. The test segments included various types of asphalt and concrete pavements. While a few segments consisted of newly paved roads, the majority exhibited varying degrees of pavement distress, such as potholes and cracks. Additionally, the test area featured multiple speed bumps and manhole covers, which could generate significant vibrations if riders did not reduce speed when passing over them, potentially causing discomfort to the cyclists.

#### 3.2.2. Test Vehicle

**Figure 10:**
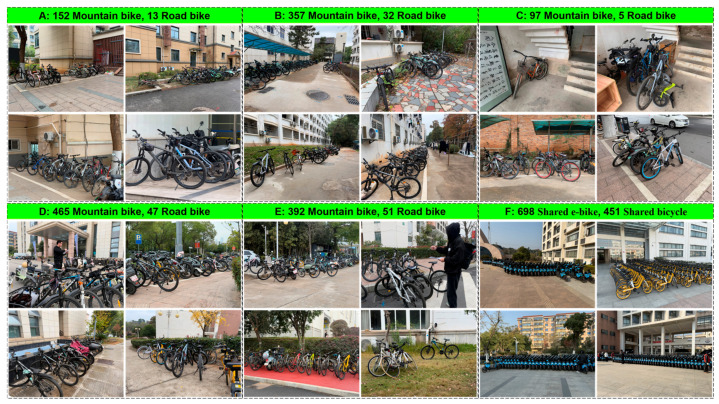
Vehicle statistics on the campus of East China Jiaotong University.

The test area was located on the campus of East China Jiaotong University. To ensure the test conditions closely aligned with actual usage scenarios, a comprehensive survey was conducted prior to the study, categorizing the existing bicycles on campus by zones. The university currently has 1463 Mountain Bikes, 148 Road Bikes, 698 Shared E-bikes (concentrated in designated parking areas, not campus-wide), and 451 Shared Bicycles (concentrated in designated parking areas, not campus-wide), totaling 2760 bicycles, as illustrated in Figure 10.

**Figure 11:**
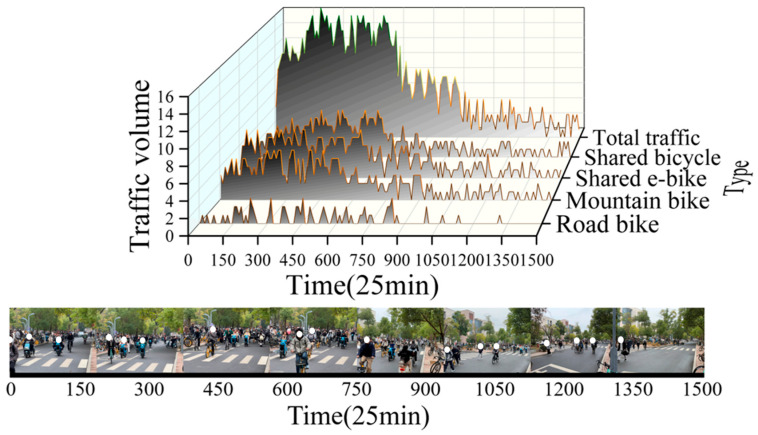
Traffic volume monitoring at East China Jiaotong University from 9:40 a.m. to 10:05 a.m.

Additionally, to obtain more realistic and accurate data on vehicle usage, this study recorded traffic flow data through time interval photography at a crossroad near the teaching buildings of East China Jiaotong University. The observation was conducted from 9:40 a.m. to 10:05 a.m., lasting for 25 min. This methodology can also be applied to urban traffic flow statistics. As illustrated in Figure 11, during this period, a total of 71 Road Bikes, 354 Mountain Bikes, 393 Shared E-bikes, and 337 Shared Bicycles passed through the intersection, amounting to 1155 vehicles in total.

**Figure 12:**
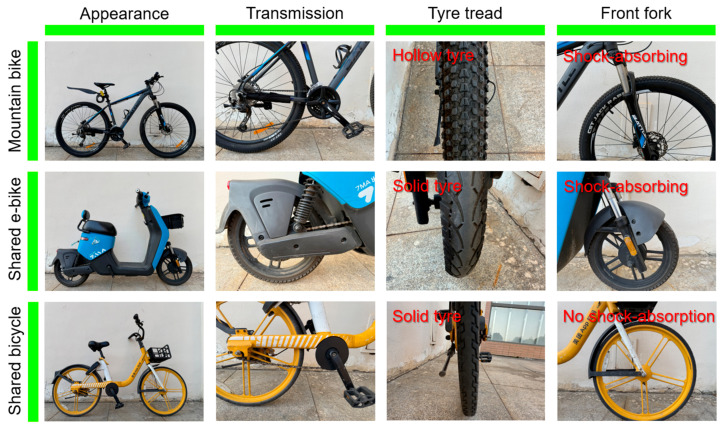
Test vehicles.

**Figure 13:**
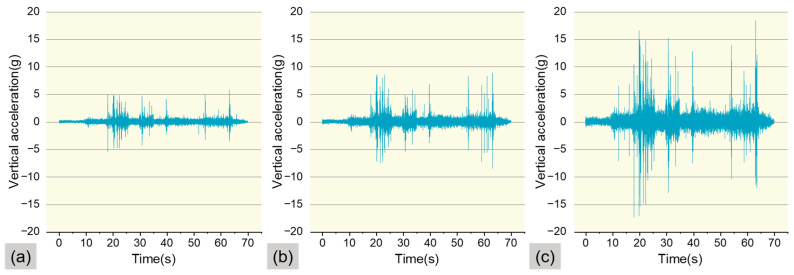
Unprocessed raw vibration signals obtained by the Riding Vibration Dynamic Testing Hardware System (Using Route 1 as an example). (**a**) Mountain Bike; (**b**) Shared E-bike; (**c**) Shared Bicycle.

Based on this data, this study selected Mountain Bike, Shared E-bike, and Shared Bicycle as the test vehicles, as shown in Figure 12. The model of the Mountain Bike is BATTLE-X5, the model of the Shared E-bike is GXDDC-HJ, and the model of the Shared Bicycle is MT-HJ. The raw, unprocessed *z*-axis data of these three vehicle types recorded by the riding vibration dynamic testing system are illustrated in Figure 13. To ensure the consistency and reliability of the experimental results, all three vehicles selected for this study were within one year of use and exhibited minimal wear. Prior to vibration testing, key components were systematically inspected and performance-verified to ensure that all vehicles were in normal working condition.

During the testing process, the tire pressure of the Mountain Bikes was maintained at 80 MPa, with measurements conducted hourly to ensure consistent pressure across all road segments. Both Shared E-bike and Shared Bicycles were equipped with solid tires, thus eliminating the influence of tire pressure. Suspension systems were activated on Mountain Bikes and Shared E-bikes, whereas Shared Bicycles lacked any suspension. Furthermore, to assess potential variability among different vehicles of the same category, vibration tests were randomly performed on three units each of Mountain Bikes, Shared E-bike, and Shared Bicycles along identical routes at uniform riding speeds. The results indicated minor variations among vehicles of the same type; however, these differences did not significantly affect the validation of the testing methodology.

### 3.3. Cycling Comfort Map for Three Types of Vehicles

This study integrates vibration acceleration data with latitude and longitude data to construct a map for assessing cycling comfort. Based on the three most frequently used types of cycling vehicles on the campus of East China Jiaotong University—Mountain Bike, Shared E-bike, and Shared Bicycle—the map categorizes cycling comfort into three main levels: green (comfortable, $a_{wv}\leq 0.8\;m/s^{2}$), yellow (uncomfortable, $0.8\;m/s^{2}<a_{wv}\leq 2.0\;m/s^{2}$), and red (very uncomfortable, $a_{wv}>2.0\;m/s^{2}$). To provide a more refined expression of comfort, the study further divides these levels into ten subcategories, as shown in Table 3. This method not only visually displays the spatial distribution of cycling comfort on roads but also provides quantitative data support for road management authorities to plan and maintain infrastructure more effectively. For example, in “red” zones, road management authorities can implement targeted maintenance or localized repairs to improve cycling comfort. Compared to traditional vibration measurement methods (such as visual inspections or subjective rider feedback), the comfort map-based evaluation approach offers significant advantages. It not only helps road management authorities scientifically assess the service level of road infrastructure but also provides cyclists with valuable references for route selection, thereby further promoting the sustainable development of bicycle transportation.

**Figure 14:**
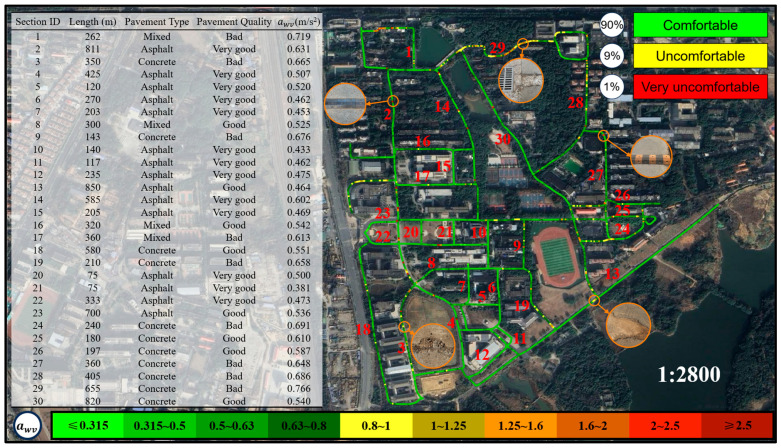
Cycling comfort map for Mountain bike.

Figure 14 illustrates the comfort map for Mountain Bike riding. According to ISO 2631-1, whole-body vibration assessment requires at least 60 s of continuous data. All road segments tested in this study met this requirement. The table on the left of Figure 14 summarizes the $a_{wv}$ values for 30 road segments, all of which are below 0.8 m/s^2^—the threshold defined by ISO for “beginning of discomfort.” The comfort map on the right side of Figure 14 visualizes the spatial variation in vibration. Based on ISO 2631-1, 90% of the routes were classified as comfortable (green), 9% as slightly uncomfortable (yellow), and 1% as clearly uncomfortable (red). Field observations indicated that discomfort in yellow zones was mainly caused by surface damage, manholes, or debris, while red zones were mostly associated with sudden structural changes such as speed bumps. This finding aligns with existing studies [10,11], which highlight that Mountain Bikes—equipped with larger tires, lower tire pressure, and front suspension systems—effectively attenuate high-frequency vibrations induced by uneven surfaces, thereby enhancing ride comfort. Additionally, pavement composition plays a crucial role in determining comfort levels [22]. Asphalt and well-maintained concrete surfaces provide a relatively smooth riding experience, whereas severely deteriorated concrete or mixed-surface roads generate pronounced vibrations, compromising comfort.

**Figure 15:**
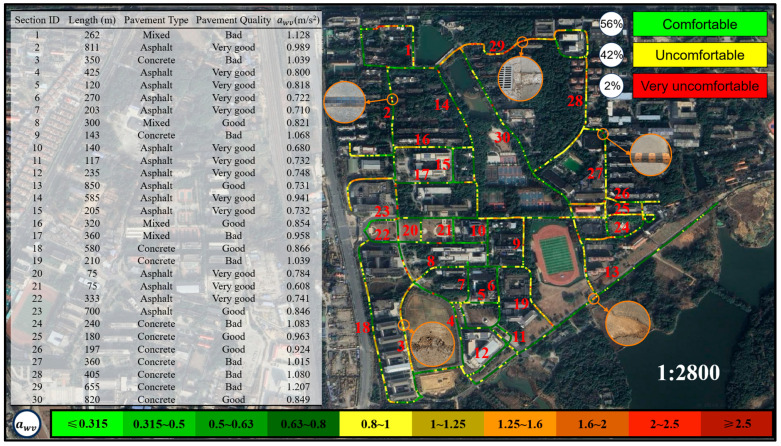
Cycling comfort map for Shared E-bike.

Figure 15 presents the cycling comfort map for Shared E-bikes. Compared to Mountain Bikes, Shared E-bikes exhibit a higher overall vibration level. Among the 30 tested routes, 8 showed vibration levels exceeding 1 m/s^2^, indicating a high likelihood of rider discomfort, while the remaining routes may still induce varying degrees of discomfort. According to the comfort map, 56% of the segments were classified as comfortable (green), 42% as uncomfortable (yellow), and 2% as highly uncomfortable (red). On well-paved asphalt or high-quality concrete surfaces, vibration levels typically ranged from 0 to 0.8 m/s^2^, ensuring a relatively smooth riding experience. However, on poorly maintained roads, vibration levels often ranged between 0.8 and 2 m/s^2^, and when passing over speed bumps, potholes, or manholes at higher speeds, the vibration intensity increased noticeably. In these cases, peak instantaneous acceleration could exceed 2 m/s^2^, significantly affecting ride comfort and potentially compromising vehicle stability.

Although the proportion of uncomfortable segments is notably higher, the overall vibration intensity remains within an acceptable range and does not reach levels that would cause severe discomfort. Vehicle mass has been shown to influence vibration levels [8,29]. Field observations suggest that while Shared E-bikes are equipped with front suspension like Mountain Bikes, their greater weight and solid rubber tires limit their shock absorption capacity. Additionally, the rigid frame structure and more upright riding posture [30] amplify impact perception when riding over rough terrain.

**Figure 16:**
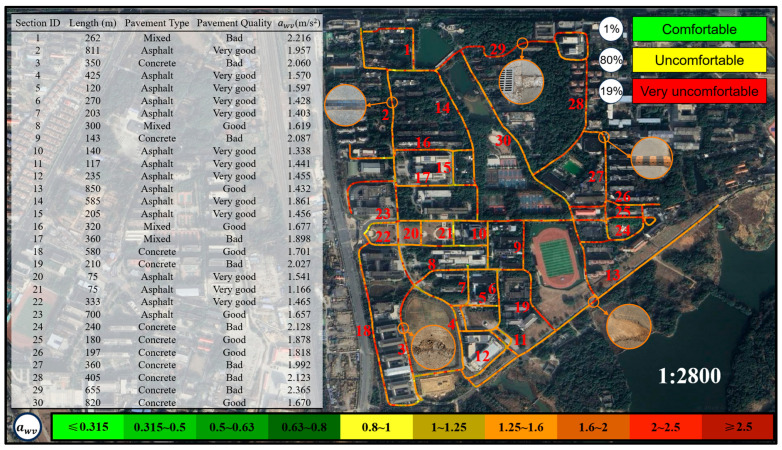
Cycling comfort map for Shared Bicycle.

Figure 16 illustrates the cycling comfort map for Shared Bicycles. Among the three types evaluated, Shared Bicycle exhibited the highest vibration levels. Across all 30 tested segments, the $a_{wv}$ exceeded 1 m/s^2^, surpassing the ISO 2631-1 threshold associated with significant discomfort. Comfort classification indicates that only 1% of the routes were comfortable (green), 80% moderately uncomfortable (yellow), and 19% highly uncomfortable (red). Even on well-maintained surfaces, vibration levels from Shared Bicycles typically exceeded 0.8 m/s^2^. On deteriorated mixed-material or concrete roads, peak accelerations often surpassed 2 m/s^2^—significantly higher than those recorded for Mountain Bikes and Shared E-bikes. These elevated vibration levels are primarily attributed to the design of Shared Bicycles: they generally feature rigid frames with no suspension systems and use solid or high-pressure tires to reduce maintenance needs, which greatly limits their ability to attenuate high-frequency vibrations.

Moreover, the upright riding posture and stiff saddles commonly found on Shared Bicycles intensify vibration transmission to the rider. As a result, vertical acceleration experienced by the body increases significantly on uneven terrain, leading to a marked decline in whole-body vibration comfort.

**Figure 17:**
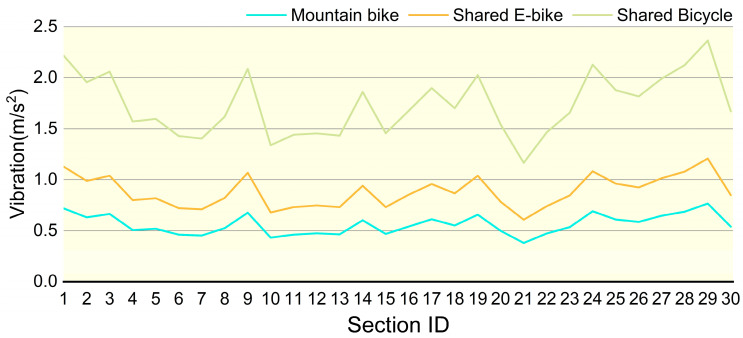
Comparison of vibration levels among three vehicles.

A comparative analysis of vibration intensities among the three bicycle types is presented in Figure 17. The results indicate that Mountain Bikes exhibit the lowest vibration intensity, attributed to their superior shock absorption capacity and enhanced tire adaptability. Shared E-bikes demonstrate moderate vibration levels; despite being equipped with front-fork suspension, their relatively high overall weight limits the effectiveness of vibration attenuation. In contrast, Shared Bicycle experiences the highest vibration intensity due to the absence of effective shock absorption mechanisms, resulting in more pronounced impacts on riders when traversing rough terrain. An integrated analysis of the cycling comfort maps from Figure 14, Figure 15 and Figure 16 reveals that Mountain Bikes maintain a comfortable riding experience on 90% of the surveyed routes. However, the proportion of comfortable segments decreases to 56% for Shared E-bike and a mere 1% for Shared Bicycle, underscoring the significant influence of bicycle type on ride comfort in addition to road surface conditions. Moreover, the elevated vibration levels of Shared Bicycles not only diminish riding comfort but may also pose safety concerns. Prolonged exposure to excessive vibrations can reduce handlebar stability, potentially increasing the difficulty of maintaining control over the bicycle in highly uneven terrain.

In previous studies, data collection primarily relied on a single accelerometer and a GPS unit. This method presented issues such as operational inconvenience, insufficient time synchronization accuracy, and an inability to support real-time monitoring. The cyclic vibration test system developed in this study effectively overcomes these limitations. Compared to traditional vibration evaluation methods, which are confined to numerical analysis, the conventional approach makes it difficult to visualize vibration intensity. Moreover, the large volume of data increases analytical complexity, and it is challenging for cyclists to intuitively perceive road comfort. By integrating Geographic Information System (GIS) technology, a cycling comfort map was constructed. This map not only visualizes vibration data, enabling cyclists to intuitively identify variations in vibration, but also incorporates synchronously recorded image data, allowing direct observation of road conditions in high-vibration areas.

## 4. Conclusions

This study developed a hardware system for cycling vibration testing, conducted practical measurements, and utilized QGIS to generate cycling comfort maps for three types of bicycles. The principal conclusions are as follows: 

1. The system accurately records three-axis vibration data (x, y, z) and synchronizes it with GPS coordinates. Its modular design enables easy maintenance and scalability, making it suitable for broader applications.

2. This study employs QGIS Desktop 3.34.12 software to introduce two methods for map creation: manual drafting and Python-based automation. Comfort maps are generated for Mountain Bikes, Shared E-bikes, and Shared Bicycles. Cycling comfort is categorized into three tiers based on vibration acceleration ($a_{wv}$) values: green (comfortable, $a_{wv}\leq 0.8\;m/s^{2}$), yellow (uncomfortable, $0.8\;m/s^{2}<a_{wv}\leq 2.0\;m/s^{2}$), and red (highly uncomfortable, $a_{wv}>2.0\;m/s^{2}$). To enhance the map’s precision, the comfort levels are further subdivided into ten distinct grades.

3. Beyond road surface conditions, vehicle type also exerts a significant influence on vibration intensity. A comparative analysis of comfort maps reveals that under identical road conditions and cycling speeds, Mountain Bikes exhibit the lowest vibration levels, ensuring a comfortable riding experience on approximately 90% of the surveyed routes. In contrast, Shared E-bikes have a discomfort rate of 42%, while Shared Bicycles record the highest vibration levels, with nearly 80% of the routes potentially causing discomfort. Consequently, although all three vehicle types are suitable for short-distance commuting, Mountain Bikes and Shared E-bikes are more advisable for prolonged rides due to their superior vibration mitigation.

4. This study developed a vibration testing system and conducted on-road experiments to collect acceleration data for evaluating whole-body vibration based on the ISO 2631-1 standard. By visualizing the vibration data through comfort maps, the method proved feasible for identifying road surface conditions and their impact on rider comfort. These maps help cyclists plan more comfortable routes based on objective vibration exposure levels and also hold potential value for road quality monitoring.

While the study validates the feasibility of this approach, it primarily focuses on objective physical vibration metrics, without fully considering inter-individual variability and intra-vehicle differences. Human sensitivity to vibration varies with physiological characteristics and riding behavior. Future work should incorporate subjective comfort data from different user groups, refine the overlapping comfort thresholds defined in ISO 2631-1, and embed individual response patterns into the vibration testing system to build a more adaptive and intelligent evaluation model. Current classification methods rely mainly on accelerometer data, which may not fully capture subjective perception. Integrating subjective assessments with vibration measurements could establish a more comprehensive framework aligned with human physiological and psychological responses.

## Figures and Tables

**Table 1:** Parameters and functions of each module.

Module Name	Parameters	Functions
Raspberry PI 5	Operating memory: 4 GB LPDDR4-3200 SDRAM; Interface: 2 × USB3.0, 2 × USB2.0; Connection: WIFI6, Bluetooth 5.0; System: Raspberry Pi OS. Manufacturer: Changsha yaomai Intelligent Technology Co., Ltd. (Changsha, China)	Responsible for executing the instructions and computations necessary for the proper operation of the system, ensuring efficient data acquisition and writing by the hardware.
Accelerometer	Model: Lis3dh; Range: ±2 g, ±4 g, ±8 g, ±16 g; Test setting range: ±16 g; Sampling frequency: 400 Hz. Manufacturer: STMicroelectronics (Geneva, Switzerland)	Acquires triaxial vibration acceleration data.
Position module	Model: LC29H; Positioning system: GPS, Beidou; Update frequency: 1 Hz; Latitude and longitude format: NMEA0183; Positioning accuracy: ±1 m. Manufacturer: Quectel (Shanghai, China)	Acquires data such as latitude, longitude, cycling speed, and cycling distance.
Camera	Pixel: 8 million. Manufacturer: Sony (Tokyo, Japan)	Performs real-time recording of the test road environment to facilitate retrospective analysis and comparison.
Operation interface	Size: 7 inches; Resolution: 1024 × 600 pixels; Principle: Touch; Interfaces: USB, HDMI, Power port. Manufacturer: MAKEROBOT (New York, NY, USA)	This system is employed during the data acquisition process to facilitate human–machine interaction, enabling test personnel to monitor in real-time and adjust equipment parameters as necessary.
Power Supply	Specifications: 12 V 5100 mAh, 12 V 5600 mAh. Manufacturer: WHEELTEC (Tampa, FL, USA)	It is utilized to power the Raspberry Pi and the display screen.

**Table 2:** Delineates the classification of vibration intensity and its potential impact on human comfort levels.

Vibration Intensity (m/s^2^)	Potential Comfort Level
Below 0.315	Very comfortable
0.315~0.63	Slight uncomfortable
0.5~1	Uncomfortable
0.8~1.6	Very uncomfortable
1.25~2.5	Quite uncomfortable
Above 2.5	Extremely uncomfortable

**Table 3:** Comfort level classification and corresponding values adopted in this study.

Vibration Intensity (m/s^2^)	Comfort Level	Line Colour (HEX)	Assignment
Below 0.315	Very comfortable	#8DFF01	1
0.315~0.5	Slight uncomfortable	#32C800	2
0.5~0.63	Slight uncomfortable or Uncomfortable	#2A9E00	3
0.63~0.8	Uncomfortable	#006813	4
0.8~1	Uncomfortable or Very uncomfortable	#FFFF47	5
1~1.25	Very uncomfortable	#B4A200	6
1.25~1.6	Very Uncomfortable or Quite uncomfortable	#FF9501	7
1.6~2	Quite uncomfortable	#C45F00	8
2~2.5	Quite uncomfortable or Extremely uncomfortable	#FF2701	9
Above 2.5	Extremely uncomfortable	#A01D00	10

## Data Availability

The data presented in this study are available on request from the corresponding author due to privacy.

## References

[1] Hongfei C., Yuxin L., Mengyuan C. (2024). How to Make Carbon Reduction Compatible with Economic Growth: An Empirical Study Based on the New Energy Demonstration City Policy. Financ. Res..

[2] Gao J., Sha A., Huang Y., Liu Z., Hu L., Jiang W., Yun D., Tong Z., Wang Z. (2019). Cycling comfort on asphalt pavement: Influence of the pavement-tyre interface on vibration. J. Clean. Prod..

[3] Hölzel C., Höchtl F., Senner V. (2012). Cycling comfort on different road surfaces. Procedia Eng..

[4] Hunt J.D., Abraham J.E. (2007). Influences on bicycle use. Transportation.

[5] Fan M., Lv J., Yu C., Guo Y., Bian Z., Yang S., Yang L., Chen Y., Huang Y., Chen B. (2019). Association Between Active Commuting and Incident Cardiovascular Diseases in Chinese: A Prospective Cohort Study. J. Am. Heart Assoc..

[6] Maizlish N., Woodcock J., Co S., Ostro B., Fanai A., Fairley D. (2013). Health cobenefits and transportation-related reductions in greenhouse gas emissions in the San Francisco Bay area. Am. J. Public Health.

[7] Budd D., Holness D.L. (2018). Raising awareness of hand-arm vibration syndrome (HAVS) using posters. Work.

[8] Olieman M., Marin-Perianu R., Marin-Perianu M. (2012). Measurement of dynamic comfort in cycling using wireless acceleration sensors. Procedia Eng..

[9] Gao J., Sha A., Huang Y., Hu L., Tong Z., Jiang W. (2018). Evaluating the cycling comfort on urban roads based on cyclists’ perception of vibration. J. Clean. Prod..

[10] Ayachi F.S., Dorey J., Guastavino C. (2015). Identifying factors of bicycle comfort: An online survey with enthusiast cyclists. Appl. Ergon..

[11] Lépine J., Champoux Y., Drouet J. (2015). The relative contribution of road bicycle components on vibration induced to the cyclist. Sports Eng..

[12] Petrone N., Giubilato F. (2013). Development of a Test Method for the Comparative Analysis of Bicycle Saddle Vibration Transmissibility. Procedia Eng..

[13] Arellana J., Saltarín M., Larrañaga A.M., González V.I., Henao C.A. (2020). Developing an urban bikeability index for different types of cyclists as a tool to prioritise bicycle infrastructure investments. Transp. Res. Part A Policy Pract..

[14] Akar G., Clifton K.J. (2009). Influence of Individual Perceptions and Bicycle Infrastructure on Decision to Bike. Transport Res. Rec..

[15] Pérez-Zuriaga A.M., Llopis-Castelló D., Just-Martínez V., Fonseca-Cabrera A.S., Alonso-Troyano C., García A. (2022). Implementation of a Low-Cost Data Acquisition System on an E-Scooter for Micromobility Research. Sensors.

[16] Zhu S., Zhu F. (2019). Cycling comfort evaluation with instrumented probe bicycle. Transp. Res. Part A Policy Pract..

[17] Segadilha A.B.P., Sanches S.D.P. (2014). Analysis of Bicycle Commuter Routes Using GPSs and GIS. Procedia-Soc. Behav. Sci..

[18] Menghini G., Carrasco N., Schüssler N., Axhausen K.W. (2010). Route choice of cyclists in Zurich. Transp. Res. Part A Policy Pract..

[19] Ambrož M. (2017). Raspberry Pi as a low-cost data acquisition system for human powered vehicles. Measurement.

[20] Vanwalleghem J., Mortier F., De Baere I., Loccufier M., Van Paepegem W. (2012). Design of an instrumented bicycle for the evaluation of bicycle dynamics and its relation with the cyclist’s comfort. Procedia Eng..

[21] Bíl M., Andrášik R., Kubeček J. (2015). How comfortable are your cycling tracks? A new method for objective bicycle vibration measurement. Transp. Res. Part C Emerg. Technol..

[22] Nuñez J.Y.M., Bisconsini D.R., Rodrigues Da Silva A.N. (2020). Combining environmental quality assessment of bicycle infrastructures with vertical acceleration measurements. Transp. Res. Part A Policy Pract..

[23] Doria A., Marconi E., Munoz L., Polanco A., Suarez D. (2021). An experimental-numerical method for the prediction of on-road comfort of city bicycles. Veh. Syst. Dyn..

[24] Ahmed T., Pirdavani A., Wets G., Janssens D. (2024). Evaluating Bicycle Path Roughness: A Comparative Study Using Smartphone and Smart Bicycle Light Sensors. Sensors.

[25] Yamanaka H., Pan X., Sanada J. (2013). Evaluation Models for Cyclists’ Perception Using Probe Bicycle System. J. East. Asia Soc. Transp. Stud..

[26] Anjum M.T., Tabassum S., Alqubaysi T., Saleemi H., Sahar U.U., Alanazi F. (2024). Accessibility enhancement of mass transit system through GIS based modeling of feeder routes. J. Urban Manag..

[27] Haery S., Mahpour A., Vafaeinejad A. (2024). Forecasting urban travel demand with geo-AI: A combination of GIS and machine learning techniques utilizing uber data in New York City. Environ. Earth Sci..

[28] (1997). Mechanical Vibration and Shock-Evaluation of Human Exposure to Whole-Body Vibration—Part 1: General Requirements.

[29] Li Z., Wang W., Liu P., Ragland D.R. (2012). Physical environments influencing bicyclists’ perception of comfort on separated and on-street bicycle facilities. Transp. Res. Part D Transp. Environ..

[30] Polanco A., Marconi E., Muñoz L., Suárez D., Doria A. Effect of Rider Posture on Bicycle Comfort. Proceedings of the ASME 2019 International Design Engineering Technical Conferences and Computers and Information in Engineering Conference.

